# Supplementary material for: Symptom severity trajectories and distresses in patients undergoing video-assisted thoracoscopic lung resection from surgery to the first post-discharge clinic visit
Source: PLoS One. 2023 Feb 22;18(2):e0281998. doi: 10.1371/journal.pone.0281998 (PMC9946218; doi:10.1371/journal.pone.0281998)
Supplement: S1 Text — (DOCX) [file pone.0281998.s001.docx]

**S1 Text.** Questionnaire on distresses used in this study

*Q1 What made you feel distressed DURING THE HOSPITALIZATION? Please circle all numbers that apply.*

1. Severity of the symptom(s) after surgery

2. Difficulties in management of the symptom(s) after surgery

3. Unable to describe the symptom(s) to the doctor

4. Burden on your partner

5. Burden on your child (children)

6. Change in your looks

7. Cost for the treatment

8. Issues related to work

9. Unable to find a staff to consult with

10. Others (_______________)

*Q2 Please describe what you would have liked to know more DURING THE HOSPITALIZATION.*

**[_____________________________________________________________________]**

*Q3 What made you feel distressed AFTER THE HOSPITAL DISCHARGE? Please circle all numbers that apply.*

1. Persistence of the symptom(s) after surgery

2. Decrease in physical strength

3. Weight loss

4. Unable to get staff’s advice as needed (in case of trouble)

5. Unable to make a decision to call the hospital

6. Health management (other than cancer at this time) in the future

7. Impact of the disease or symptom(s) with daily life

8. Date for return to work

9. Burden on your partner

10. Burden on your child (children)

11. Others　(__________)

*Q4 Please describe what you would have liked to know more AT THE TIME OF HOSPITAL DISCHARGE.*

[_____________________________________________________________________]

*Q5 Please rate the following statements on a 0-10 scale, 0 being the lowest (strongly disagree) and 10 being the highest (strongly agree).*

*A. The MDASI survey in this study was useful for my self-management.*

*B. The MDASI survey in this study was easy to input.*
