# Supplementary figures and images for: Symptom severity trajectories and distresses in patients undergoing video-assisted thoracoscopic lung resection from surgery to the first post-discharge clinic visit
Source: PLoS One. 2023 Feb 22;18(2):e0281998. doi: 10.1371/journal.pone.0281998 (PMC9946218; doi:10.1371/journal.pone.0281998)

**S2 Figure.** Clinical and pathological TNM in patients with diagnosis of lung cancer

**
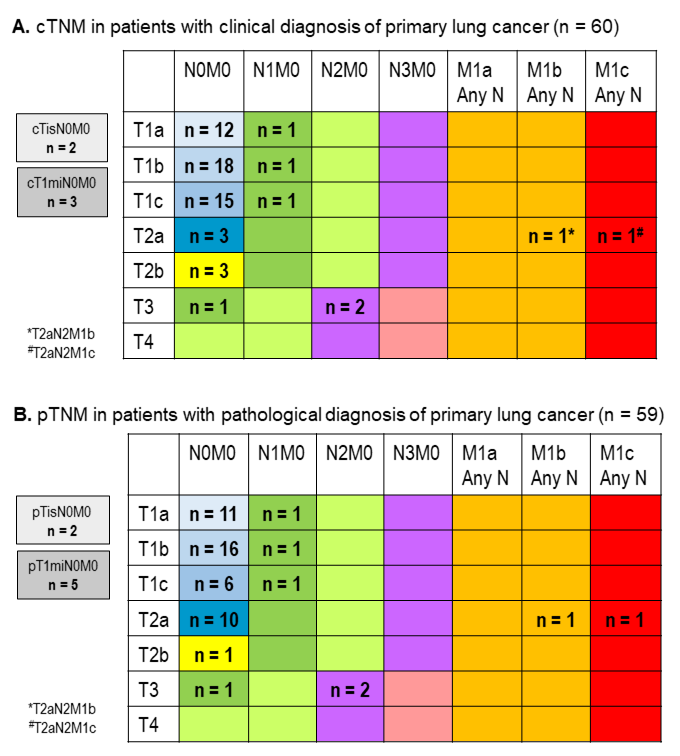
**

Supplement: S1 Fig — (DOCX) [file pone.0281998.s002.docx]
