# Supplementary material for: Symptom severity trajectories and distresses in patients undergoing video-assisted thoracoscopic lung resection from surgery to the first post-discharge clinic visit
Source: PLoS One. 2023 Feb 22;18(2):e0281998. doi: 10.1371/journal.pone.0281998 (PMC9946218; doi:10.1371/journal.pone.0281998)
Supplement: S2 Fig — (DOCX) [file pone.0281998.s003.docx]

**S5 Figure.** Summary of the patients’ opinions regarding usefulness and feasibility of the MDASI survey (response to the Q5 in the questionnaire used in this study).

**
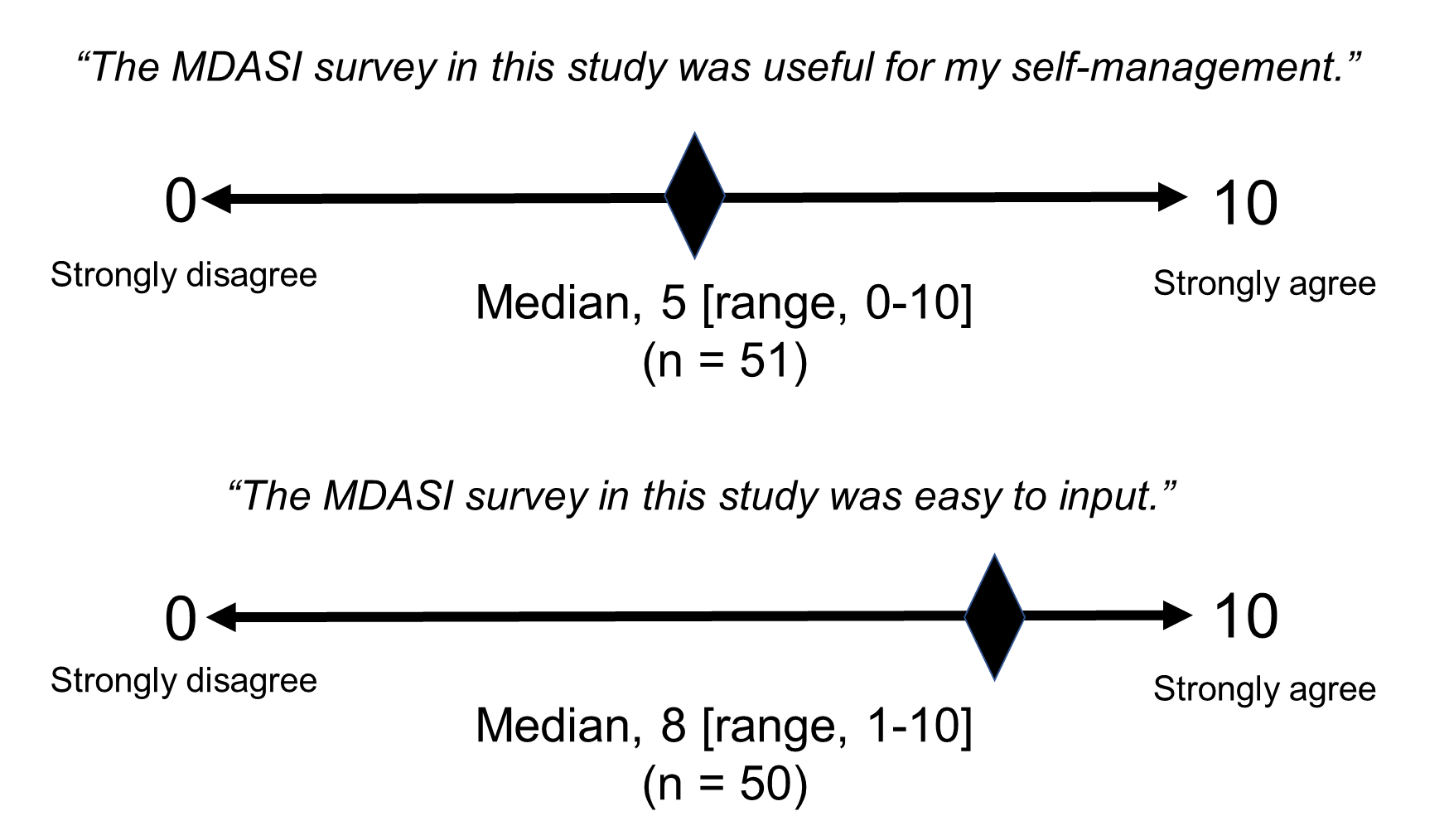
**
