# Supplementary material for: Symptom severity trajectories and distresses in patients undergoing video-assisted thoracoscopic lung resection from surgery to the first post-discharge clinic visit
Source: PLoS One. 2023 Feb 22;18(2):e0281998. doi: 10.1371/journal.pone.0281998 (PMC9946218; doi:10.1371/journal.pone.0281998)
Supplement: S1 Table — (DOCX) [file pone.0281998.s004.docx]

**S3 Table.** Summary of symptom scores for 13 core symptoms and 6 interferences with daily life

**A. Core symptoms**

| **Symptoms** | **Symptom score** | **Symptomatic* patients, (%)** |
| --- | --- | --- |
| Pain | **2.26** | 75 (100%) |
| Disturbed sleep | **1.71** | 70 (93.3%) |
| Shortness of breath | **1.41** | 64 (85.3%) |
| Drowsiness | **1.36** | 71 (94.7%) |
| Fatigue | **1.34** | 66 (88.0%) |
| Numbness and tingling | **1.20** | 61 (81.3%) |
| Dry mouth | 1.17 | 70 (93.3%) |
| Distress | 1.00 | 59 (78.7%) |
| Lack of appetite | 0.83 | 63 (84.0%) |
| Sadness | 0.70 | 48 (75.0%) |
| Remembering things | 0.31 | 42 (56.0%) |
| Nausea | 0.26 | 44 (58.7%) |
| Vomiting | 0.20 | 33 (44.0%) |

*A patient was considered to be symptomatic when they reported symptom severity of ≥ 1 at any timepoint during the study period.

**B. Interferences with daily life**

| **Symptom interference** | **Symptom score** | **Symptomatic* patients, (%)** |
| --- | --- | --- |
| General activity | **1.91** | 67 (89.3%) |
| Enjoyment of life | **1.69** | 62 (82.7%) |
| Work | **1.59** | 57 (76.0%) |
| Walking | **1.25** | 63 (84.0%) |
| Mood | **1.30** | 63 (84.0%) |
| Relations with other people | **0.78** | 47 (62.7%) |

*A patient was considered symptomatic when they reported interference level of ≥1 at any timepoint during the study period.
