# Supplementary material for: Symptom severity trajectories and distresses in patients undergoing video-assisted thoracoscopic lung resection from surgery to the first post-discharge clinic visit
Source: PLoS One. 2023 Feb 22;18(2):e0281998. doi: 10.1371/journal.pone.0281998 (PMC9946218; doi:10.1371/journal.pone.0281998)
Supplement: S2 Table — (DOCX) [file pone.0281998.s005.docx]

**S4 Table.** Bayesian information criterion for models of symptom severity trajectories calculated using the joinpoint analysis

**A. The six most severe core symptoms**

| Symptom | Bayesian information criterion for a joinpoint model of symptom severity trajectories | | | |
| --- | --- | --- | --- | --- |
|  | 0 joinpoints | 1 joinpoint | 2 joinpoints | 3 joinpoints |
| Pain | 1.63 | **-0.20** | -0.06 | 0.17 |
| Disturbed sleep | 1.13 | 0.06 | **-0.92** | -0.66 |
| Shortness of breath | -0.08 | -0.53 | -0.57 | **-0.62** |
| Fatigue | 1.06 | **-1.20** | -1.17 | -0.93 |
| Drowsiness | 1.24 | **0.09** | 0.38 | 0.66 |
| Numbness and tingling | 0.33 | **-0.76** | -0.71 | -0.55 |

**B. Interferences with daily life**

| Symptom interference | Bayesian information criterion for a joinpoint model of trajectories for level of interference | | | |
| --- | --- | --- | --- | --- |
|  | 0 joinpoints | 1 joinpoint | 2 joinpoints | 3 joinpoints |
| General activity | 0.96 | **-1.18** | -1.01 | -0.79 |
| Enjoyment of life | 0.54 | **-1.49** | -1.46 | -1.23 |
| Work | 0.25 | -0.42 | **-0.79** | -0.57 |
| Walking | 1.14 | **-0.51** | -0.43 | -0.16 |
| Mood | 1.01 | **-1.13** | -0.98 | -0.70 |
| Relations with other people | -0.23 | **-1.39** | -1.30 | -1.29 |
