# Supplementary material for: Symptom severity trajectories and distresses in patients undergoing video-assisted thoracoscopic lung resection from surgery to the first post-discharge clinic visit
Source: PLoS One. 2023 Feb 22;18(2):e0281998. doi: 10.1371/journal.pone.0281998 (PMC9946218; doi:10.1371/journal.pone.0281998)
Supplement: Attachment — Submitted filename: Response_to_the_reviewers_ver_2.3.docx [file pone.0281998.s006.docx]

April 25th, 2022

Editorial Board

*PLOS ONE*

Dear Editors:

Please find enclosed our revised manuscript entitled “**Symptom severity trajectories and distresses in patients undergoing video-assisted thoracoscopic lung resection from surgery to the first post-discharge clinic visit**” (PONE-D-22-03682). We have revised the manuscript based on the reviewers’ comments. We have addressed the reviewers’ concern below in a point-by-point manner, with our responses appearing in ***italics***.

We are extremely grateful to the Editors and the Reviewers for their careful review and helpful suggestions. We hope that the revised manuscript meets the expectations of the Editors and Reviewers and is now suitable for publication in ***PLOS ONE***.

Sincerely,

***Tomohito Saito, MD, PhD***

Department of Thoracic Surgery, Kansai Medical University Hospital

2-3-1, Shinmachi, Hirakata, Osaka 573-1191, Japan

Tel: +81-72-804-0101; Fax: +81-72-804-2865

E-mail: saitotom@hirakata.kmu.ac.jp

*AUTHORS’ RESPONSES TO COMMENTS FROM REVIEWERS*

Re: PONE-D-22-03682　“Symptom severity trajectories and distresses in patients undergoing video-assisted thoracoscopic lung resection from surgery to the first post-discharge clinic visit”

Reviewer #1:

The authors focus an important issue in evaluation of PROs in thoracic surgery; however, the results of this trial do justify only a vague conclusion. I have several concerns that are listed below:

1. The sample size is small and the study population is too heterogeneous.

*[Authors’ response]*

***In response to the Reviewer’s comment, we have revised the description of the study’s limitations in the Discussion section as follows:***

***“This study has some limitations. First, our results were based on single-institution data and a small sample size. We acknowledge the possibility that patient characteristics and clinical practices influenced our results.” [Page 33, Lines 524–526 of Marked Copy]***

***“Second, our study population had heterogenous clinical and pathological characteristics. Patients with metastatic lung cancer/benign pathology and those undergoing sublobar resection were included in our study, which could result in heterogeneity in the extent of pulmonary resection and lymph node dissection. This could impact postoperative symptoms. Additionally, 9 of 60 patients with a clinical diagnosis of lung cancer had a pathological confirmation preoperatively. Postoperative symptoms, such as disturbed sleep, distress, mood, and pain, could be affected by not only the clinical diagnosis itself, but also the type of clinical diagnosis (i.e., pathologically-confirmed or not). Thus, adjustment for the clinical diagnosis and its type should be considered in future studies.” [Page 34, Lines 534–543 of Marked Copy]***

Reviewer #1:

2. There are no data about patients with suffered on chronic pain and had possibly pain medication before surgery.

*[Authors’ response]*

***In response to the Reviewer’s comment, we have revised Table 1. We have also added the following description to the Result section:***

***“Eight (10.7%) patients had received pain medication for their chronic pain before surgery.” [Pages 11-12, Lines 263–264 of Marked Copy]***

Reviewer #1:

3. There are no clear data about the histopathological tumour status of the patient (TNM) pre and postoperatively. All these factors could influence symptom severity trajectories and distress.

*[Authors’ response]*

***In response to the Reviewer’s comment, we have revised Table 1. We have also added a supplemental figure (S3 Figure) describing the preoperative and postoperative TNM statuses. Furthermore, we have added the following statement to describe the preoperative pathological diagnosis and (postoperative) pathological stage:***

***“Clinical stages 0-II lung cancer was the most common preoperative clinical diagnosis, accounting for 80.0% (60 of 75 patients). Likewise, pathological stages 0-II lung cancer was the most common postoperative pathological diagnosis, accounting for 78.7% (59 of 75 patients). Detailed information on the patients’ clinical and pathological TNM findings is shown in S3 Figure. Of note, a pathological diagnosis of lung cancer was made preoperatively in 9 patients.” [Page 12, Lines 265–270 of Marked Copy]***

Reviewer #1:

4. It is not clear in which amount the numbers of ports affect the main endpoint.

*[Authors’ response]*

***As shown in the Table 4 in the original manuscript (Table 2 in the Marked Copy), no association between the number of ports and the main endpoint was detected. In response to the Reviewer’s comment, we have added the following statement to clarify this point:***

***“Of note, the number of ports and the type of regional anesthesia (EA or PVB) showed no statistically significant association with recovery of pain to a mild level.” [Page 21, Lines 413–415 of Marked Copy]***

Reviewer #1:

5. The clinical pathway is faulty and do not correspond to current ERAS pathways.

*[Authors’ response]*

***In response to the Reviewer’s comment, we have added the following statement to the Discussion section:***

***“Forth, the clinical pathway employed during the study period did not correspond to up-to-date Enhanced Recovery After Surgery (ERAS) protocols. As implementation of ERAS pathways has been shown to be associated with improved patient outcomes [39], our results should be validated as part of a clinical pathway that conforms to recent ERAS guidelines [40].” [Page 34, Lines 550–554 of Marked Copy]***

Reviewer #1:

6. Pain therapy algorithm is described; however, no data are available about the amount of pain medication or additional pain medication the patients had really preserved.

*[Authors’ response]*

***In response to the Reviewer’s comment, we have revised Table 1 to clarify the amount of pain medication used and the instances of rescue pain medication administration.***

Reviewer #1:

7. There are too many tables, could be reduced to the essential.

*[Authors’ response]*

***In response to the Reviewer’s comment, we have removed two tables (Tables 2 and 3 in the original version) from the main manuscript and now provide them in the form of supporting information as S4 Table and S5 Table, respectively.***

Reviewer #2:

This manuscript is well written with a good English. It is technically sound, and the data support the conclusions. The authors made all data underlying the findings in their manuscript fully available and the statistical analysis is performed appropriately.

However I still have some questions to the authors:

1- Do you think knowing the diagnosis before surgery (as some of your patients did and some not), more specifically knowing that they have cancer or not, or even having a primary or secondary cancer, would effect the postoperative symptoms of the patients, such as disturbed sleep, drowsiness, distress, mood, and even pain. I think you should be commenting on these in discussion.

***In response to the Reviewer’s comment, we have revised the Discussion section as follows:***

***“Second, our study population had heterogenous clinical and pathological characteristics. Patients with metastatic lung cancer/benign pathology and those undergoing sublobar resection were included in our study, which could result in heterogeneity in the extent of pulmonary resection and lymph node dissection. This could impact postoperative symptoms. Additionally, 9 of 60 patients with a clinical diagnosis of lung cancer had a pathological confirmation preoperatively. Postoperative symptoms, such as disturbed sleep, distress, mood, and pain, could be affected by not only the clinical diagnosis itself, but also the type of clinical diagnosis (i.e., pathologically-confirmed or not). Thus, adjustment for the clinical diagnosis and its type should be considered in future studies.” [Page 34, Lines 534–543 of Marked Copy]***

Reviewer #2:

2- I think you should emphasize if there was any difference between the groups having a paravertebral or an epidural block.

*[Authors’ response]*

***In response to the Reviewer’s comment, we have revised Table 2 of the Marked Copy. We have also added the following description to the Result section:***

***“Of note, the number of ports and the type of regional anesthesia (EA or PVB) showed no statistically significant association with recovery of pain to a mild level.” [Page 21, Lines 413–415 of Marked Copy]***

Reviewer #2:

3- I think you should also be giving information about the details of the surgical technique, as primary cancer patients probably had lymph node dissection also, apart from lobectomy, secondary cancer patients might had it too or not, and patients with a benign lesion probably had no lymph node dissection, and these details might have effected some postoperative symptoms also.

*[Authors’ response]*

***In response to the Reviewer’s comment, we have added the following statement to the Method section:***

***“For patients undergoing lobectomy for primary lung cancer, lobe-specific lymph node dissection was routinely performed. For those undergoing lobectomy for metastatic lung tumor, either lobe-specific or hilar lymph node dissection was selected at the surgeon’s discretion. Hilar lymph node dissection was accompanied with segmentectomy, whereas no lymph node dissection was performed in conjunction with wedge resection.” [Pages 7–8, Lines 166–171 of Marked Copy]***

***Further, in response to the Editorial Office’s request, we have added S6 Figure to provide the data regarding feasibility of the MDASI survey. We have corrected the numbers of the patients who responded the questionnaire [Page 34, Lines 548-550 of Marked Copy].***

***Additionally, we recognized that the description in Table 1 required the following corrections:***

***Surgery type***

***Lobectomy	54 (72.0%)***

***Segmentectomy	1 (1.3%)***

***Wedge resection 20 (26.7%)***

***We sincerely apologize for any confusion caused.***
