# Supplementary material for: Symptom severity trajectories and distresses in patients undergoing video-assisted thoracoscopic lung resection from surgery to the first post-discharge clinic visit
Source: PLoS One. 2023 Feb 22;18(2):e0281998. doi: 10.1371/journal.pone.0281998 (PMC9946218; doi:10.1371/journal.pone.0281998)
Supplement: Attachment — Submitted filename: Response_to_the_reviewers_R2_ver_2022-12-19.docx [file pone.0281998.s007.docx]

*AUTHORS’ RESPONSES TO COMMENTS FROM REVIEWERS*

Re: PONE-D-22-03682R1　“Symptom severity trajectories and distresses in patients undergoing video-assisted thoracoscopic lung resection from surgery to the first post-discharge clinic visit”

Reviewer #1:

I have no further comments, all comments have been addressed. I accept the revised manuscript for publication.

*[Authors’ response]*

***We appreciate the reviewer’s careful review and helpful suggestions that have benefitted our manuscript considerably.***

Reviewer #2:

This is an interesting article that identifies characteristics regarding postoperative quality of life in patients who have undergone VATS lung resection.

As I have some questions or comments, please answer these issues.

Page 19, Line 370; The 20 day cumulative recovery to a mild level for pain … were 55,8%, …

I think the recovery rate of postoperative pain 55.8% is too low, what do you think about it?

*[Authors’ response]*

***We agree with the reviewer that the 20-day (3-week) cumulative rate of recovery of pain to mild severity in our study was relatively lower than that noted in previous studies as shown in Table 3. Possible explanations for this difference might include the difference in patient characteristics and in the pain management protocol.***

***In response to the reviewer’s comment, we have included the above information in the Discussion section as follows:***

***“The 20-day (3-week) cumulative rate of recovery of pain to mild severity in our study was relatively lower than that reported in previous studies [16,17], for which the possible explanations include the difference in patient characteristics and in the pain management protocol.” [Pages 34–35, Lines 538–541 of the Marked Copy]***

Page 20, Line 385; Further, among pain severity on day 4, …

You collected data on pain from postoperative days 1 to day 4, and stated that the value on day 4 was optimal for determining the cutoff value, but if you had collected data on day 5 as well, wouldn't it be even more predictive? Do you think it is right?

*[Authors’ response]*

***In terms of predictive power, the pain level on days 5, 6, and 7 seemed better than that on day 4 (AUC, 0.755, 0.842, and 0.823 vs. 0.723, respectively). However, we selected day 4 for further analysis as we identified day 4 as the earliest timepoint to predict pain recovery at the time of the first post-discharge clinic visit (Figure 5). This was based on the concept that earlier prediction of pain recovery would give us more chances for intervention to improve the patients’ outcomes.***

***In response to the reviewer’s comment, we have revised the Methods section to clarify the reason why we focused on the pain level at the earliest timepoint to predict pain recovery as follows:***

***“As a secondary analysis, the accuracy of pain severity on days 1, 2, 3, 4, 5, 6, and 7 for predicting recovery of pain to a mild level at the first post-discharge clinic visit was determined using area under the receiver operating characteristic (ROC) curves. In this analysis, the cut-off level of pain at the earliest timepoint to predict pain recovery at the time of the first post-discharge clinic visit was also determined by the ROC curve. This was based on the concept that earlier prediction of pain recovery would give us more chances for intervention to improve the patients’ outcomes.” [Pages 10-11, Lines 236-244 of the Marked Copy]***

***“Univariate analysis was used to evaluate the cut-off level of pain at the earliest timepoint determined by the ROC curve” [Page 11, Lines 245-247 of the Marked Copy]***

***Furthermore, we have also revised Figure 5 and the Results section as follows:***

***“The ROC curves of pain severity level on days 1, 2, 3, 4, 5, 6, and 7 for predicting recovery of pain to mild level at the time of the first post-discharge clinic visit are shown in Fig 5. The area under the curve (AUC) of pain severity level on days 1, 2, 3, 4, 5, 6, and 7 were 0.502, 0.549, 0.626, 0.723, 0.755, 0.842, and 0.823 (P = 0.98, P = 0.48, P = 0.069, P = 0.001, P < 0.001, P < 0.001, and P < 0.001), respectively. Thus, day 4 was identified as the earliest timepoint to predict pain recovery at the time of the first post-discharge clinic visit.” [Page 20, Lines 385–391 of the Marked Copy]***

Page 21, Line 401; Fig. 5;

This curve suggests that day 3 is a better indicator than days 1 and 2, and day 4 is a better indicator than day 3. Does this also suggest that day 5 (or day 6...) may be a better predictor than day 4?

*[Authors’ response]*

***As mentioned in our response to your comment above, in terms of predictive power, the pain level on days 5, 6, and 7 seemed better than that on day 4 (AUC, 0.755, 0.842, and 0.823 vs. 0.723, respectively). However, we selected day 4 for further analysis as we identified day 4 as the earliest timepoint to predict pain recovery at the time of the first post-discharge clinic visit (Figure 5). This was based on the concept that earlier prediction of pain recovery would give us more chances for intervention to improve the patients’ outcomes.***

***In response to the reviewer’s comment, we have revised the Methods section to clarify the reason why we focused on the pain level at the earliest timepoint to predict pain recovery as follows:***

***“As a secondary analysis, the accuracy of pain severity on days 1, 2, 3, 4, 5, 6, and 7 for predicting recovery of pain to a mild level at the first post-discharge clinic visit was determined using area under the receiver operating characteristic (ROC) curves. In this analysis, the cut-off level of pain at the earliest timepoint to predict pain recovery at the time of the first post-discharge clinic visit was also determined by the ROC curve. This was based on the concept that earlier prediction of pain recovery would give us more chances for intervention to improve the patients’ outcomes.” [Pages 10-11, Lines 236-244 of the Marked Copy]***

***“Univariate analysis was used to evaluate the cut-off level of pain at the earliest timepoint determined by the ROC curve” [Page 11, Lines 245-247 of the Marked Copy]***

***Further, we have also revised Figure 5 and the Results section as follows:***

***“The ROC curves of pain severity level on days 1, 2, 3, 4, 5, 6, and 7 for predicting recovery of pain to mild level at the time of the first post-discharge clinic visit are shown in Fig 5. The area under the curve (AUC) of pain severity level on days 1, 2, 3, 4, 5, 6, and 7 were 0.502, 0.549, 0.626, 0.723, 0.755, 0.842, and 0.823 (P = 0.98, P = 0.48, P = 0.069, P = 0.001, P < 0.001, P < 0.001, and P < 0.001), respectively. Thus, day 4 was identified as the earliest timepoint to predict pain recovery at the time of the first post-discharge clinic visit.” [Page 19, Lines 385–391 of the Marked Copy]***

Page 32, Line 507;

How do you think the absolute value of pain intensity affected this result? Did patients with stronger pain in the early postoperative period rebound more during the chronic period?

Does this association between pain on day 4 and chronic pain relate to so-called "pain memory"?

*[Authors’ response]*

***As shown in Figure 5, pain levels on days 4-7, not those on days 1-3, were associated with pain recovery to a mild level at the time of the first post-discharge clinic visit. This association may further support the concept of “pain memory,” where peripheral and central pain sensitization play a pertinent role in pain chronification.***

***In response to the reviewer’s comment, we have also revised the Discussion and References sections as follows:***

***“Interestingly, chronic pain and memory share the anatomical sites of synaptic plasticity such as the limbic-cortical pathways [36]. Further, neuroimaging studies have suggested that the chronification of pain may result from persistence of pain memory via spatiotemporal reorganization of the neocortex [36].” [Pages 33–34, Lines 513–516 of the Marked Copy]***

***“This may indicate the possible association of delayed recovery of acute postsurgical pain with persistence of pain memory.” [Pages 34, Lines 518–520 of the Marked Copy]***

***[36] McCarberg B, Peppin J. Pain Pathways and Nervous System Plasticity: Learning and Memory in Pain. Pain Med. 2019;20: 2421-2437. doi: 10.1093/pm/pnz017. PMID: 30865778. [Page 42, Lines 707–709 of the Marked Copy]***

***Additionally, we recognized that the citation of Table numbers in the text required the following corrections:***

***(Table 3) [Page 28, Line 472 of the Marked Copy]***

***(Table 2) [Page 34, Line 518 of the Marked Copy]***

***We sincerely apologize for this confusion.***
